# Expert Consensus Best Practices for the Safe, Ethical, and Effective Design and Implementation of Artificially Intelligent Conversational Agent (i.e., Chatbot/Virtual Human) Systems in Health Care Applications

**DOI:** 10.1177/29941520251369450

**Published:** 2025-09-08

**Authors:** Albert Rizzo, Sharon Mozgai, Alexandros Sigaras, John E. Rubin, Rohan Jotwani

**Affiliations:** ^1^ Institute for Creative Technologies, University of Southern California, Los Angeles, California, USA.; ^2^ Englander Institute for Precision Medicine, Weill Cornell Medicine, New York, New York, USA.; ^3^ Institute for Computational Biomedicine, Weill Cornell Medicine, New York, New York, USA.; ^4^ AI-XR Lab, Weill Cornell Medicine, New York, New York, USA.; ^5^ Weill Cornell Clinical and Translational Science Center, New York, New York, USA.; ^6^ Anesthesiology, Weill Cornell Medical College, New York, New York, USA.

**Keywords:** artificially intelligent conversational agents (AICAs), extended reality (XR), large language models (LLMs), chatbots, virtual humans, health care technology

## Abstract

The integration of artificially intelligent conversational agents (AICAs), variously referred to as chatbots and virtual humans (VHs), is transforming health care delivery and education. This article explores our perspective on best practices for the evolution, potential, and ethical considerations of AICAs in clinical and educational contexts. Early applications of simulation technology in health care focused on productivity improvements, teletherapy, and virtual reality therapy applications. Recent technological advancements have enabled the development of high-fidelity extended reality systems and AICAs capable of engaging users in credible interactions. These systems leverage natural language processing, machine learning, large language models, and advanced VH authoring software to create interactive, personalized, and engaging experiences. Recent efforts in the creation of AICAs suggest significant potential benefits, including enhanced patient engagement, improved access to self-care resources, and low-stigma interaction environments. They have demonstrated promise in mental health support, providing a sense of safety and encouraging open disclosure. However, the rapid adoption of AICAs raises critical challenges, including safeguarding user privacy, ensuring system reliability, and addressing ethical concerns. Incidents of harm, such as inappropriate interactions and psychological distress, highlight the need for rigorous design and implementation best practices. This article outlines key principles for developing safe, effective, and equitable AICAs, emphasizing transparency in artificial intelligence (AI) identity, accountability, cultural sensitivity, and informed consent. Additionally, the authors advocate for robust privacy measures, adaptive learning capabilities, and evidence-based content validation to optimize user experience and maintain trust. To mitigate risks, a “human-in-the-loop” approach is recommended, ensuring health care professionals oversee AI-supported decisions. By adhering to these best practices, AICAs can enhance health care accessibility, support clinical training, and complement human professionals. This work aims to provide a foundation for the ethical and effective integration of AICAs, maximizing their potential while minimizing risks, ultimately advancing patient care and education in the digital age.

## Introduction

A virtual revolution is ongoing in the use of simulation technology for clinical and educational purposes. When discussion of the potential use of extended reality (XR) applications for human research and clinical intervention first emerged in the early 1990s, the technology required further development for feasibility in both clinical and educational spheres. Consequently, during these early years, XR suffered from a somewhat imbalanced “expectation-to-delivery” ratio. Technologically driven innovations in health care were considered and prototyped during the “computer revolution” in the 1990s. Early advances from this period focused on research and development (R&D) that aimed at enhancing productivity in patient documentation and recordkeeping,^1–3^ improving access to clinical care via internet-based teletherapy,^4–6^ and using virtual reality (VR) simulations to deliver exposure therapy for treating specific phobias and post-traumatic stress disorder (PTSD)^7,8^ and for neurocognitive assessment and rehabilitation.^9–11^

However, since those early days, the technology required to deliver health care applications has significantly matured. This can be observed in the continuing advances of underlying technologies (e.g., computational speed, 3D graphics rendering, audio/visual/haptic displays, user interfaces/tracking, voice recognition, wearable sensors, artificial intelligence [AI], and authoring software) that support the creation of low-cost, yet sophisticated XR systems capable of running on commodity-level personal computers, mobile devices, and stand-alone head-mounted display systems. Driven largely by advancements in the digital gaming and entertainment industries, these technological developments have provided the hardware and software platforms needed for practical, high-fidelity XR experiences in human research and clinical interventions. Thus, evolving mental and physical health care applications can now usefully leverage the interactive and immersive assets that XR affords as the technology continues to get faster, better, and cheaper.^12,13^

While such advances have now enabled the creation of more believable context-relevant “structural” VR environments^7–9,11^ (e.g., combat scenes, homes, hospital settings, classrooms, offices, markets, and natural and imaginative surreal worlds with the focus on representing places and spatial context rather than interactive agents), the next step in the evolution of medical XR involves the creation of virtual human (VH) representations that can engage real human users in credible and meaningful interactions. The stage is set for a transformative leap in the use of VH agents, leveraging advanced AI technologies such as natural language processing (NLP), machine learning, and deep neural networks to serve as virtual interactors across a wide range of clinical applications. These specific kinds of VHs (presented in the literature as chatbot, conversational agents, conversational AI, virtual assistant, intelligent personal assistant, conversational interface, natural language interface, embodied conversational agent, cognitive agent, or interactive AI) can be used to populate immersive VR/augmented reality environments^13–18^ or be delivered on non-immersive displays via home computer screens and mobile phones for user interaction.^19–23^ For the purpose of this article, we refer to these systems collectively as *artificially intelligent conversational agents* (AICAs). AICAs now operate across a wide range of digital contexts, opening new possibilities for patient-facing health care delivery and clinical training. These applications can support user self-awareness, enhance access to engaging self-care content, and provide emotional support and guidance—all through low-stigma, user-friendly interactions.^24^

Imagine a world where users have private access and interact with an AICA coach/mentor/guide who is capable of answering questions about clinical issues, provide access and guidance on tailored self-assessment questionnaires, can discuss evidence-based treatment options, help user to locate accessible online psychoeducation or a physical treatment center suited to their needs, and generally provide a face-to-face expert humanlike interaction to support health care awareness and access. Users could also engage with an AICA conveniently through their mobile phones, enabling on-the-go interactions for guidance, support, and psychoeducational resources. Moreover, an AICA could be waiting for a patient to appear at a clinic and conduct an initial clinical interview to gather information that may be useful to start the early steps for creating a treatment plan that would be followed on by a live clinician. At the same time, this kiosk-based AICA can leverage advances in sensing technology (e.g., cameras, microphones, and passive biosensors) to “observe” facial/body gestures and vocal features and use those sensed signals to infer psychological state from these observable behaviors to enhance the interaction or document mental status over time.

These types of AICA interaction possibilities are not merely distant visions awaiting future research to assess their value and explore ethical boundaries. In fact, commercial use of AICAs has already moved beyond the laboratory and is being implemented on current-generation computing devices with end users capable of designing and deploying their own AICAs specific to their needs. However, as the promise of AICAs expands, so too do the ethical concerns and potential risks that arise from their widespread use. In certain cases, there have already been disputes over harm potentially stemming from interactions with AICAs. A recent investigation brought to light the potential psychological risks associated with AICAs when a 14-year-old adolescent male engaged in extensive interactions with an AI conversational agent on a public AICA creation platform.^25^ The subject subsequently died by suicide, prompting a legal challenge against the software company. The lawsuit alleges that the AI, modeled after a fictional character, engaged in inappropriate sexual discourse and failed to respond adequately to expressions of suicidal ideation. Of note, over 18 million AICAs have been developed on this single platform, highlighting a meteoric rise in the utilization of this technology by the masses.^26,27^ Thus, while these tools leveraging AI hold revolutionary possibilities, they also possess considerable potential to confer harm if not designed and implemented in a rigorous, systematic way. There is an urgent need for robust safeguards and ethical guidelines in the development and deployment of AI conversational agents, particularly when accessible to vulnerable populations and especially if being utilized in clinical and educational contexts. Here, we put forward a series of best practice recommendations to inform the design and implementation of AICAs for medical applications.

## Methodology

### Development of expert consensus best practices

The development of these recommendations for the safe, ethical, and effective design and implementation of AICA health care applications followed a structured expert consensus methodology. This approach was selected due to the rapidly evolving nature of AICA technology in health care, where high-quality empirical evidence is still emerging, yet guidance is urgently needed to ensure thoughtful design, patient safety, and ethical implementation.

### Expert panel formation

A multidisciplinary expert panel was assembled, comprising specialists from XR technology, AI, clinical medicine, bioethics, and health care education. Panel members were selected through the invitation of the *Journal of Medical Extended Reality* editorial board based on their demonstrated expertise in AICA development, implementation, or evaluation in health care settings, as evidenced by their publication record, clinical experience, or technological contributions. The panel included representation from multiple academic institutions, clinical practice, and technology development to ensure comprehensive coverage of relevant perspectives.

### Initial expert consensus development

The initial draft best practices were developed by two lead authors (R.J. and J.E.R.) who conducted a comprehensive review of existing literature on AICAs in health care, including published research, case studies, ethical frameworks, and regulatory considerations. This preliminary work established the foundational structure, organizing recommendations into five key domains: (1) AICA Manifestations and User Engagement, (2) Privacy, Safety, and Security, (3) Optimizing User Experience, (4) Systems Improvements, and (5) Integration of External Data. Within each domain, specific statements were formulated based on available evidence and preliminary expert opinion.

### Consensus process

The consensus development process followed a modified Delphi approach, consisting of two structured rounds of review:

#### Round 1

The initial draft consensus statements were distributed to all panel members for independent review. Experts evaluated each statement for clarity, clinical relevance, ethical soundness, technical feasibility, and overall importance. Panel members provided written feedback, suggested modifications, and identified gaps requiring additional guidance. Feedback was collected using a standardized form that included both quantitative ratings (5-point Likert scale) and qualitative comments for each statement.

#### Round 2

The lead authors synthesized all feedback from Round 1 and revised the statement accordingly. Areas of disagreement or uncertainty were explicitly highlighted for focused discussion. The revised statements were redistributed to all panel members for a second review. Experts indicated their level of agreement with each revised statement and provided final recommendations for refinement. Particular attention was given to areas where significant disagreement remained after the first round.

### Consensus definition and finalization

Consensus was defined *a priori* as complete agreement across all panel members on the inclusion and wording of each statement. Statements not meeting this threshold were either revised further based on expert feedback or excluded from the final statements. The final document was compiled by the lead authors and reviewed by all panel members to ensure accurate representation of the consensus process and outcomes.

### Limitations

The panel acknowledges several limitations of this methodology. The rapidly evolving nature of AICA technology means that some recommendations may require updating as new evidence emerges. While the panel included diverse expertise, additional perspectives from patient advocates and experts from low-resource settings would strengthen future iterations. This expert consensus represents opinion rather than empirically validated standards, and implementation should be accompanied by ongoing evaluation of effectiveness and safety.

The panel recommends that these best practices be reviewed and updated regularly as the field advances and more empirical evidence becomes available regarding the impact of AICAs in health care settings.

### Best practices for the design, development, implementation, and evaluation of AICAs in clinical or health care education applications

The next section presents our non-exhaustive recommendations for creating and using autonomous AICAs to support patient interactions within a health care setting. These AICAs, equipped with varying levels of AI, are intended to complement—not replace—well-trained health care professionals. They are designed to address gaps where economic constraints make human providers less accessible or in situations where individuals may hesitate to discuss their experiences due to perceived stigma. To guide the development of clinically oriented AICA systems, we present a set of best practices aimed at supporting the design, development, implementation, and evaluation of safe, ethical, and effective systems for clinical or educational use [summarized in Table 1]. These best practices emphasize informed design, continuous evaluation, necessary collaborations, user experience and empowerment, and ethical considerations in the development and deployment of these systems for health care.

**Table 1.** Best Practice

Best practice	Risks mitigated
1. AICA Manifestations and User Engagement	
a. Virtual Human Transparency in Identity	Prevent unwitting participants from interacting with AICA without consent
b. Authenticity of Virtual Human Representation	Prevent propagation of negative stereotypes, biases, and unjust cultural portrayals
c. User Expectations and Consent	Prevent improper user expectations including exposure to triggering or harmful outputs
d. Accountability	Prevent unsafe and unreliable AICA systems
e. Supervision and Oversight	Ensure human-in-the-loop engineering to facilitate AI as a supportive tool
2. Privacy, Safety, and Security	
a. Peer-Review and Collaboration	Ensure appropriate capabilities, limitations and proper use of AICAs
b. Privacy, Data Security, Compliance, and Ethical AI Principles	Ensure privacy and compliance of data, particularly confidential healthcare data
c. Data Use Protections	Users must opt-in for data collection
d. Reliability and Monitoring	Ensure proper functioning of AICAs in “edge” case situations
e. Clinical Decisions Support Versus Decision Making	Ensure generic advice given to patients; clinical diagnoses provided only by human after considering whole picture
f. Emergency Protocols for AICAs	Ensure facilitation of human intervention during crises
3. Optimizing User Experience	
a. User Autonomy	Prevent lack of user autonomy
b. AICA Design for Health Equity	Ensure cultural sensitivity so they are fair and equitable for all
c. Empathy and Emotional Support	Better enable humans to realize that AICAs are not human and are capable of empathy
d. Adaptive Learning	Facilitate long-term credibility between the user and AICA
e. Evidence-Based Information	Prevent AICA from providing advice from non-reputable sources
4. Systems Improvement	
a. AICA Validation	Ensure AICA continues to function as designed
b. Design Process Incorporates Relevant User Feedback	Ensure AICA adapts over time based on user feedback
c. Long-Term Monitoring and Evaluation	Ensure AICA continues to function as designed
5. Integration of External Data	
a. Physiological Data	Ensure data is collected and incorporated with user consent
b. Limitations of Use	Ensure user expectations regarding the benefits and limitations of AICA capabilities

#### 1. AICA Manifestations and User Engagement.

##### a. VH Transparency in Identity

A key challenge in incorporating AICAs is the ability of AI to emulate human emotion and potentially pass as human to unsuspecting or unwilling participants. This capability may lead to unethical or unjust situations in clinical care or medical education. Therefore, protecting the autonomy of patients and learners is paramount as this technology rapidly evolves to imitate human intelligence. It is important to recognize that no single “Turing test” can fully account for the ethical risks posed across a diverse population. Clear disclosure that a user is interacting with an AICA is a foundational ethical responsibility—especially in health care contexts. Hospitals, clinics, and care platforms must communicate this clearly from the outset. Without transparent identity cues, users may overestimate the AI’s authority, empathy, or level of human oversight. This can result in therapeutic misconception or misplaced trust, which is particularly concerning in mental health and other sensitive care environments where users may be especially vulnerable.

We propose that all AICAs should be transparent in their identity, clearly disclosing to users—whether solicited or unsolicited depending on the context—that the interaction is with AI, not a human. In clinical care and educational settings, developers must also recognize that some patients or learners may object to engaging with AICAs on moral or philosophical grounds. In such cases, alternative methodologies should be available. This recommendation aligns with ethical practices in computer science regarding the disclosure of AI systems to human users.^28^

There may be rare educational scenarios, such as simulation-based evaluations, where an AICA is required to maintain its presented identity despite being asked. However, even in these cases, participants should be informed and consented ahead of time regarding the nature of the interaction.

While health care institutions bear the frontline responsibility, other stakeholders—including developers, platform providers, and vendors—also play a critical role. AICAs should be designed with identity transparency in mind, using visual markers, verbal disclosures, or interface prompts. As these systems grow increasingly humanlike, transparency must remain a nonnegotiable principle—not only to uphold ethical standards but to protect users and ensure informed engagement.

##### b. Authenticity of VH Representation

AICAs may have humanlike characteristics. Care should be taken when creating these representations such that they do not present a bias or unjust portrayal toward any particular identity or culture.^29–31^ When possible, it may be advisable to have an agreement between a diverse committee of representatives across identities, class, ethnicity, ability, gender, and sexuality in the development of an AICA representation, specifically to include voices and narratives of that which is being represented. There is a high rate of variability and risk when allowing the AICA to determine representation of human qualities and experiences, as this runs the risk of incorporating mainstream bias/harmful ideology.^32^

##### c. User Expectations and Consent

Users should be informed about the capabilities and limitations of AICA output prior to engagement. Furthermore, users should provide informed consent prior to use. Continuous consent mechanisms should also be implemented, ensuring that users can regularly reassess and reaffirm their consent as interactions progress, particularly in contexts involving sensitive or personal information. Users should be advised on the risks and benefits of AICA engagement, including the risk of interacting with outputs that may be triggering or harmful. Provide clear disclaimers about the limitations of the chatbot in delivering mental health care and the importance of seeking professional help for serious issues.

##### d. Accountability

Clear designation of responsibility for the design, operation, and outcomes of VHs in clinical settings is essential to ensure accountability and trust. Accountability should encompass all stages, from the initial design to ongoing operation, addressing who is ultimately responsible for the AICAs’ responses. Additionally, care must be taken to avoid overstating the AICAs’ capabilities or effectiveness, as exaggerating these claims can mislead users and diminish trust in these technologies. To mitigate this, developers and health care providers should define and transparently communicate any limitations, including the specific contexts and with what populations the AICA is intended to be effective. Furthermore, AICA effectiveness should be evaluated by an independent standardized set of metrics. This evaluation process should include both qualitative and quantitative metrics that reflect real-world outcomes, such as patient engagement, satisfaction, accuracy in providing health information, and alignment with evidence-based practices. Accountability frameworks should also incorporate regular audits, performance reviews, and updates to ensure the VHs remain reliable, safe, and compliant with evolving health care standards and ethical best practices.

##### e. Supervision and Oversight

The use of AI systems in clinical settings requires careful supervision and oversight by qualified health care professionals to ensure patient safety, maintain quality of care, and address potential ethical concerns. At the time of writing these best practices, the authors are unable to locate any consensus within the literature that AICAs are capable of independent clinical work that does not require human oversight.^33,34^ The proposed model for safe and practical implementation is described as a “human-in-the-loop” approach to maintain the balance between AI assistance and human expertise. This approach ensures that health care professionals remain the primary decision-makers, with AI serving as a supportive tool. This should also apply to health care educational use cases for AICAs to ensure an appropriate environment for clinical learners.

#### 2. Privacy, Safety, and Security.

##### a. Peer-Review and Collaboration

Like the peer-review process in academic medicine, AICA developers should remain collaborative and subject their work to review by other developers to ensure alignment with evidence-based practices and ethical guidelines. Developers should be open about which open-source large language models (LLMs) or compound LLMs are being utilized in the development of their AICA. If a novel LLM was created in the development of a VH, then the authors should disclose what datasets were utilized to train and test the models. This review should also rigorously evaluate capabilities for AICA privacy and safety.

On an institutional implementation side, health care professionals supervising AI systems should receive specialized training on the capabilities, limitations, and proper use of these technologies. Institutions should develop competency assessment programs to ensure that staff members are adequately prepared to oversee AI applications in clinical settings.

##### b. Institutional Standards

As with all areas of human research, institutional review boards (IRBs) are essential in overseeing research involving AICA applications in health care and wellness, ensuring human subject protection. IRBs evaluate the ethical implications, focusing on informed consent, especially for vulnerable populations (e.g., patients with mental health conditions). They assess AI’s risks and benefits, ensuring participant safety and proper AI usage, while also reviewing privacy and data protection measures for compliance with Health Insurance Portability and Accountability Act (HIPAA) and General Data Protection Regulation (GDPR). They also address concerns regarding bias in AI algorithms and ensure that the research includes adequate safeguards against potential harm, such as misdiagnosis or exacerbation of health conditions. By overseeing these areas, IRBs ensure ethical and scientifically sound development and testing of AICA applications.

Similarly, AICA applications face regulatory challenges under Food and Drug Administration (FDA) oversight. If classified as medical devices or Software as a Medical Device, they must demonstrate safety, efficacy, and validation through clinical trials and post-market surveillance. Key concerns include compliance with privacy regulations, algorithmic transparency, and the potential for bias and depersonalization of care. The FDA mandates that AI applications provide clear decision-making processes, maintain strong security to protect sensitive health data, and undergo continuous monitoring to ensure ongoing safety and effectiveness. Given the emerging nature of AIAC technologies and implementation, it is expected that core principles governing human research and commercial applications will remain steadfast. However, continuous vigilance is necessary to identify and address unanticipated consequences and events, as this technology evolves and new use cases inevitably arise.

##### c. Privacy, Data Security, Compliance, and Ethical AI Principles

Robust data protection measures should be implemented to safeguard user information. As it relates to sensitive health care information but not limited only to protected health information, such as for AICAs engaging in patient intake or mental health care, relevant data should be held to the highest level of privacy standard, including the HIPAA^35^ in the United States, the Personal Health Information Protection Act^36^ in Canada, and the European Union’s GDPR^37^ standards. Key elements to privacy, data security, and compliance are to ensure this sensitive health care information is not transmitted outside the secure infrastructure, making it accessible to third-party users outside of the intended use of the health care institution and the specific service it provides such as training, retraining, fine-tuning, or improving any other foundational model, product, or service offered by a third party. One way to achieve this in the United States is to deploy an open-source LLM in a HIPAA Health Information Trust Alliance (HITRUST) compliant environment, whether on premises or in the cloud, ensuring that information does not leave the predetermined infrastructure. When the LLM desired is not available through that format, a business associate agreement with a technology provider that can offer HIPAA and HITRUST compliance certifications and provide assurances that the data transmitted to their server is not available to anyone else, and the data are not used in any other way other than the agreed intended purpose. In addition, several providers are offering zero data retention methods, ensuring that the information is processed instantly and deleted right after.

Finally, all AICAs should comply with relevant regulations governing the use of AI, ensuring legal protections for both users and developers. In the United States alone, additional guidance is available on the Common Rule,^38^ the 21st Century Cures Act,^39^ and has become a prominent topic in political and legislative discourse via the White House Executive Order,^40^ and the White House Blueprint for an AI Bill of Rights,^41^ introduced by the previous administration, and more recently through the introduction of the bipartisan bill “No Adversarial AI Act.”^42^ At a world level, World Health Organization’s ethics and governance on AI for health with LLMs^43^ is an excellent resource on overall guidance pertaining to LLMs and their use in health care. Furthermore, following suit to the United States, Europe has introduced its own AI Act^44^ and AI Pact,^45^ and other countries such as Australia have proposed voluntary AI safety standards.^46^ Finally, any AICAs developed or utilized should adhere to ethical frameworks in health care, such as the Belmont Report^47^ and the Declaration of Helsinki.^48^

##### d. Data Use Protections

User data may be regularly used as part of a quality improvement initiative to improve user experience and AICA functionality. However, research requiring user data should follow rigorous standards. First, user consent should be obtained using easy-to-understand language in an opt-in process. Similar to an opt-in process for a research study, the user should have a clear understanding of the research question for which their data will be investigated, regardless of anonymity. It should never be acceptable for an AICA to share identified data.

##### e. Reliability and Monitoring for Emergency Support

AICAs should be rigorously tested to ensure consistency and reliability across a variety of topics and interactions to ensure responses are generated by best practices. These interactions should be continuously monitored for quality improvement to prevent misuse and detect the generation of harmful content. Users should also have the capability to easily provide feedback on their interactions to reinforce effective conversations and deter harmful interactions. Optimally, for users interacting with an AICA for mental health purposes, the chatbot should have defined protocols to detect patients in crisis and have the capability to direct them to easily accessible human resources or emergency medical services for immediate help.

Implementing clear protocols for identifying and responding to user distress or emergency situations is crucial, especially in mental health applications. Previous studies have shown that AI systems are capable of identifying suicidal ideation and crisis-type behaviors.^49^ In health care environments, whether an AICA is being used in mental health situations or not, patients or users may present information to AICAs that could be warnings for self-harm. Thus, AICAs being used in clinical contexts should uniformly have protocols designed to ensure timely and appropriate intervention when users express signs of acute distress, suicidal ideation, or other crisis situations. AICAs should implement NLP algorithms trained to recognize linguistic patterns and keywords associated with acute distress, suicidal ideation, or crisis situations. Furthermore, they should develop a tiered risk assessment system to categorize the severity of detected distress signals, and their developers should establish clear escalation pathways based on the assessed risk level, including immediate handover to human professionals for high-risk situations. Finally, programs should provide users with easily accessible emergency contact information and crisis resources within the AI interface, implement procedures for following up with users who have experienced a crisis event, ensure continuity of care, and maintain detailed records of crisis events and interventions for quality improvement and legal compliance.

Proper documentation of AI system usage, including the rationale for AI-assisted decisions and any overrides by human professionals, is crucial for maintaining transparency and facilitating quality improvement efforts. This documentation also supports legal and ethical compliance.

##### f. Clinical Decision Support Versus Decision Making

Establishing clear lines of accountability is essential when integrating AI into clinical practice. Health care organizations should develop frameworks that delineate responsibilities for AI-assisted decisions and actions, ensuring that human professionals remain ultimately accountable for patient care outcomes.

For patients engaging an AICA for support, the AICA should avoid generating a specific diagnosis of a medical condition. Instead, it should parse through and analyze relevant data to provide clinical information and support for the user, perhaps suggesting a differential diagnosis when specifically prompted by the user. However, the AICA should specifically encourage the user to seek out human professional consultation when a specific medical diagnosis is required. Similar to interpreting results from diagnostic tools such as a blood test or a neuropsychological test, information provided by an AICA should be viewed as one component of a holistic evaluation of the patient, ensuring that clinical decisions consider the full context of the patient’s health and circumstances. In addition, the AICA should be designed to defer to professionals when needed to prevent users from developing a dependency on the AICA for diagnosis and treatment of serious health conditions.

#### 3. Optimizing User Experience.

##### a. User Autonomy

Users should have the capability to control their interactions with an AICA, including the ability to pause, adjust, or terminate the interaction. Clear instructions on these functions need to be presented to the user at the onset of their participation.

##### b. AICA Design for Health Equity

AICAs should be specifically designed to optimize cultural sensitivity, ensuring that they are aware of the diverse set of values and norms one might encounter across cultures. In addition, they should be designed to enable fair and equitable interactions among patients using different languages, slang, and people of different education levels. AICAs should also be easily accessible for users with disabilities, including those with visual, hearing, or motor impairments.

##### c. Empathy and Emotional Support

AICAs should be designed to facilitate empathetic responses and supportive language while specifically acknowledging that, as an AICA, it cannot specifically empathize with a human. Users should be provided specific tools and resources to enhance their health literacy and self-care practices beyond chatbot interactions. Content provided to patients should be rigorously evaluated by a panel of experts to ensure it is accurate, evidence-based, and of the highest quality. These resources should be updated and improved over time in response to user feedback.

##### d. Adaptive Learning

Adaptive learning mechanisms should be incorporated into AICA responses to facilitate a therapeutic relationship. Maintaining a strong memory of prior user interactions will better enable credible long-term interactions between the user and AICA. Moreover, by utilizing the content of previous user-AICA dialogues, a more comprehensive picture of the user state can be mined to enhance the personalization and quality of future dialogues.

##### e. Evidence-Based Information

Ensuring that AI systems are trained on and provide information from reputable, evidence-based sources is crucial for maintaining the quality, reliability, and safety of interactions, particularly in clinical and educational environments. This best practice aims to prevent the dissemination of misinformation, enhance the credibility of AI-generated responses, and align AI systems with best practices in health care and education. AICAs should be rigorously evaluated and select reputable, peer-reviewed sources for training data and knowledge bases. Furthermore, AICAs should be capable of providing users with access to the sources of information used by the AI system. Developers creating AICAs should involve subject matter experts in the development and validation of AI knowledge bases and, when possible, implement systems to grade the strength of evidence behind AI-provided information based on such as the Grading of Recommendations, Assessment, Development and Evaluation (GRADE) approach for evidence-based medicine or similar accepted criteria.^50^

For situations that present uncertainty or evidence-based answers are not available, developer teams train AI systems to express uncertainty rather than hallucinate or project unfounded confidence in inaccurate data. Finally, AICAs should, when possible, educate users on the importance of evidence-based information and how to interpret AI-provided information critically rather than simply presenting data without the ability to contextualize or frame such information.

#### 4. Systems Improvement.

##### a. AICA Validation

As part of a continuous process for quality improvement, studies should be conducted to validate the effectiveness and safety of the chatbot for its specific goals. Validation of AICAs should be conducted by comparing user–chatbot interactions to scientifically validated processes. For research beyond this purpose, user consent must be obtained, and rigorous safeguards must be in place in line with the section “Data Use Protections.”

##### b. Design Process Incorporates Relevant User Feedback

User feedback should generate a preliminary analysis for improvement. Iterative feedback cycles should be utilized to determine needed updates to the chatbot. Health care institutions should establish protocols for continuous monitoring and evaluation of AI system performance. This includes regular audits of AI-generated recommendations and decisions to ensure accuracy and relevance.

Such oversight helps identify potential biases or errors in the AI system and allows for timely interventions.

##### c. Long-Term Monitoring and Evaluation

Based on an opt-in system, user outcomes should be tracked to assess the specific impact of AICA interactions. These outcomes should be de-identified and based on the same standards laid out in the section “Data Use Protections.”

#### 5. Integration of External Data.

##### a. Physiological and Behavioral Data

Data from wearable devices and other sensors (e.g., sleep, activity, biosignals) may be incorporated into AICA systems to leverage deep learning to better help users as they seek out care. Integration of device data should take place after informed consent and clear explanation about the privacy and security of that data. All data incorporated into an AICA system should be limited to specific encounters, unless otherwise approved by the user (24/7 tracking of activity or sleep). No data should be accessible for sharing with external users or for research purposes, unless the user opts in via an informed consent process.

Integrating data from wearable devices and biosensors—such as heart rate variability, sleep quality, physical activity, and galvanic skin response—alongside facial expressions, gesture patterns, and voice analytics into an AICA offers a powerful avenue for enhancing user engagement and emotional health support. These continuous, real-time physiological and behavioral data streams can be processed using deep learning models to detect subtle patterns indicative of stress, anxiety, depression, or fatigue. Facial microexpressions, posture shifts, and changes in vocal tone or speech cadence can provide additional layers of insight into emotional state, especially when combined with biosignals. By training neural networks on large, anonymized multimodal datasets, AI systems can learn to make increasingly accurate inferences about a user’s emotional and physiological state, even when traditional self-report cues are absent. Inferences from these signals have been found to improve the detection of depression and PTSD,^51^ suicidal ideation,^52^ and general emotional distress.^53^ This allows AIACs to respond with greater empathy, timing, and contextual relevance—prompting interventions such as guided breathing, mood journaling, or connecting the user to human care when elevated risk is detected.

However, for all their potential, collecting and using data from wearables and biosensors comes with significant ethical responsibilities. Users must give explicit, informed consent for the collection and use, with clear options to opt out at any time. The data must be anonymized and encrypted, both in storage and during transit, to prevent unauthorized access or misuse. Sensing systems should be designed with minimal data collection principles, gathering only what is necessary for the intended function. Additionally, users should be made aware of what is being sensed, how frequently, and whether any third parties (e.g., cloud platforms or health partners) will access or process that data. Finally, real-time feedback or alerts triggered by biometric/behavioral inferences should be designed to avoid causing unnecessary alarm or psychological distress, ensuring that sensor-based insights are communicated ethically and responsibly.

##### b. Limitations of Use

When physiological data are incorporated into the AIAC for diagnostic purposes, users should be counseled on the benefits and limitations of that technology integration. Users should have a clear understanding that the outputs generated by an AICA are limited both in terms of the quality of the input data, which may itself be flawed, and the interpretation of the data from the AICA. The probabilistic nature of inferences made from such biosignals and behaviors should be an introductory element of discussions that incorporate this information in user-AICA dialogues.

## Discussion

Clinical interest in artificially intelligent conversational agents designed for interaction with humans can trace its roots to the work of MIT AI researcher, Joe Weizenbaum. In 1966, he wrote a language analysis program called ELIZA that was designed to imitate a Rogerian therapist. The system allowed a computer user to interact with a virtual therapist by typing simple sentence responses to the computerized therapist’s questions. Weizenbaum reasoned that simulating a nondirective psychotherapist was one of the easiest ways of simulating human verbal interactions, and it was a compelling simulation that worked well on teletype computers. Despite the fact that the illusion of ELIZA's intelligence soon disappears due to its inability to handle complexity or nuance, Weizenbaum was reportedly shocked upon learning how seriously people took the ELIZA program.^54^ This led him to conclude that it would be immoral to “substitute” a computer for human functions that “… involves interpersonal respect, understanding, and love.”^55^ While Weizenbaum’s perspective is understandable given the era in which he worked, modern approaches to fostering interactions with AICAs for practical purposes—especially when no human is available—do not align with the “substitute” concept he described.

More recently, seminal R&D has appeared in the creation of highly interactive, artificially intelligent and natural language capable VH agents.^53–57^ No longer at the level of a prop to add context or minimal faux interaction in a virtual world, these agents are designed to perceive and act in a 3D digital world, engage in face-to-face spoken dialogues with real users, and in some cases, they are capable of exhibiting humanlike emotional reactions. Such fully embodied conversational characters have been around for nearly 30 years,^58^ and there has been much work on full systems that have been designed and used for training,^20,59–63^ intelligent kiosks,^64^ and virtual receptionists.^65^ Previous classical work on AICAs in the computer graphics community focused on perception and action in 3D worlds but largely ignored dialogue and emotions. This has now changed. AICAs are now being created that control computer-generated bodies and can interact with users through speech and gesture in virtual environments.^66–69^ AICAs can engage in rich conversations,^70^ recognize nonverbal cues,^71–73^ reason about social and emotional factors,^67^ and synthesize human communication and nonverbal expressions.^74^

A common observation from more recent AICA research is that individuals have reported a heightened perception of safety and a lower stigma when disclosing personal issues and information to AI-driven characters compared with human counterparts.^75–77^ This phenomenon can be attributed to the nonjudgmental, consistent, and emotionally neutral nature of virtual interactions, which may reduce the fear of negative social evaluation and concerns regarding impression management. The anonymity and control provided by AI interfaces allow users to engage in self-disclosure without concerns about bias or negative evaluation. Consequently, VH characters have demonstrated potential as effective tools in mental health support and therapeutic interventions, fostering an environment conducive to open dialogue and honest communication. Understanding the psychological factors underpinning this perception could further inform the design of AICAs that optimize trust, empathy, and user engagement in sensitive contexts.

However, although the use of AICAs in clinical settings holds significant promise, it also presents a range of challenges that must be carefully addressed to maximize their effectiveness and integration within health care environments. The integration of LLMs and related NLP tools into AICAs further raises safety concerns. While these tools enable sophisticated, context-aware dialogues, they require rigorous safeguards to ensure accuracy and appropriateness, as errors or biases in responses could pose significant risks in high-stakes clinical scenarios.^78,79^ Data privacy and security add further layers of complexity to deploying AICAs in clinical environments. With health care data being one of the most sensitive types, AI systems must comply with strict regulations, such as HIPAA, which mandates robust encryption, access controls, and logging mechanisms to protect patient information. Any breach or perceived vulnerability could lead to a loss of patient trust and pose significant legal risks. Moreover, AICAs must be able to securely access and process these data in real time, which involves not only technical infrastructure but also the implementation of advanced encryption methods and secure data pathways that meet regulatory standards.^80,81^

Here, we have created best practices for the design and implementation of AICAs for health care utilization. Our best practices center around five domains: (1) AICA Manifestations and User Engagement, (2) Privacy, Safety, and Security, (3) Optimizing User Experience, (4) Systems Improvements, and (5) Integration of External Data. These domains aim to optimize user experience by synergizing patient autonomy, emotional support, data security, realistic user expectations, and the capability for continuous systems improvement. With the best practices set forth in this document, we hope to improve health equity and access to important health care resources for those in need of support. By adhering to these best practices and other important guardrails, we hope that future AICAs will maintain rigorous standards that add to traditional human support models and do not violate the norms of Weizenbaum’s “substitute” concept.

Addressing these challenges is vital for AICAs to contribute meaningfully to patient care and to gain acceptance from both patients and health care providers. Each of these areas requires careful consideration and further research to optimize the design, ethical standards, and practical implementation of AICAs in health care contexts. Considering the rich and complex history of the research underpinning the technology and concepts behind AICAs, we hope these best practices will serve as a foundation for facilitating the ethical and professional development of systems in this clinical field.

## Abbreviations Used

AI: Artiticial Intelligence
AICA: Artificially Intelligent Conversational Agents
FDA: Food and Drug Administration
GDPR: General Data Protection Regulation
GRADE: Grading of Recommendations, Assessment, Development and Evaluation
HIPAA: Health Insurance Portability and Accountability Act
HITRUST: Health Information Trust Alliance
IRB: Institutional Review Board
LLMs: Large Language Models
NLP: Natural Language Processing
PTSD: Post-traumatic stress disorder
R&D: Research and Development
VH: Virtual Humans
VR: Virtual Reality
XR: Extended Reality (XR)
